# Supplementary material for: A bacterial riboswitch class for the thiamin precursor HMP-PP employs a terminator-embedded aptamer
Source: eLife. 2019 Apr 5;8:e45210. doi: 10.7554/eLife.45210 (PMC6478431; doi:10.7554/eLife.45210)
Supplement: Figure 2—source data 1. [file elife-45210-fig2-data1.docx]

|  | **Fluorescence Values** | | |  |
| --- | --- | --- | --- | --- |
| **HMP (μM)** | Replicate 1 | Replicate 2 | Replicate 3 | Average |
| 0 | 625 | 696 | 927 | 749 |
| 0.032 | 795 | 851 | 782 | 809 |
| 0.1 | 934 | 872 | 741 | 849 |
| 0.32 | 982 | 1024 | 656 | 887 |
| 1 | 2575 | 1956 | 2378 | 2303 |
| 3.2 | 5396 | 4465 | 6045 | 5302 |
| 10 | 6118 | 7823 | 6938 | 6960 |
| 32 | 7797 | 8545 | 8473 | 8272 |

Fluorescence values used to generate the plot in Fig. 2C. See the legend to Fig. 2C and the Materials and methods section for additional details.
