# Supplementary material for: A bacterial riboswitch class for the thiamin precursor HMP-PP employs a terminator-embedded aptamer
Source: eLife. 2019 Apr 5;8:e45210. doi: 10.7554/eLife.45210 (PMC6478431; doi:10.7554/eLife.45210)
Supplement: Supplementary file 3. [file elife-45210-supp3.docx]

**Supplemental File 3**

**A bacterial riboswitch class for the thiamin precursor HMP-PP employs a terminator-embedded aptamer**

**Ruben M. Atilho^1†^, Gayan Mirihana Arachchilage^2†^, Etienne B. Greenlee^3^, Kirsten M. Knecht^1^, Ronald R. Breaker^1-3^***

^1^Department of Molecular Biophysics and Biochemistry, ^2^Howard Hughes Medical Institute, ^3^Department of Molecular, Cellular and Developmental Biology, Yale University, P.O. Box 208103, New Haven, CT 06520-8103, USA.

^†^These authors contributed equally to this work

*For correspondence: [ronald.breaker@yale.edu](mailto:ronald.breaker@yale.edu)

Phone: 203 432-9389

**Supplementary Table 1.** Sequences of synthetic DNAs used in this study.

| Name | Sequence (5′ to 3′) | Annotation | |
| --- | --- | --- | --- |
| thiS1 | AAAAAAGGCCACACGAAAAAATACACCCAAGGTGGTGTACCCCTTCGTGTGACCTTCACACTCTGTCCTATAGTGAGTCGTATTA | The template for transcription of *C.* sp. Maddingley 66 *thiS* RNA | |
| thiS2 | ACGAAAAAATACACCCAAGGTGGTGTACCCCTTCGTGTGACCTTCACACTCTGTCCTATAGTGAGTCGTATTA | The template for transcription of *C.* sp. Maddingley 54 *thiS* RNA | |
| thiS3 | CGAAAAAATACACCCAAGGTGGTGTACCCCTTCGTGTGACCTTCACACTCTGTCCTATAGTGAGTCGTATTA | The template for transcription of *C.* sp. Maddingley 53 *thiS* RNA | |
| thiS4 | GAAAAAATACACCCAAGGTGGTGTACCCCTTCGTGTGACCTTCACACTCTGTCCTATAGTGAGTCGTATTA | The template for transcription of *C.* sp. Maddingley 52 *thiS* RNA | |
| thiS5 | AATTTTACACCCAAGGTGGTGTACCCCTTCGTGTGACCTTCACACTCTGTCCTATAGTGAGTCGTATTA | The template for transcription of M3 50 *thiS* RNA RNA |  |
| thiS6 | AAATTTTACACCCAAGGTGGTGTACCCCTTCGTGTGACCTTCACACTCTGTCCTATAGTGAGTCGTATTA | The template for transcription of M3 51 *thiS* RNA RNA |  |
| thiS7 | GAAATTTTACACCCAAGGTGGTGTACCCCTTCGTGTGACCTTCACACTCTGTCCTATAGTGAGTCGTATTA | The template for transcription of M3 52 *thiS* RNA RNA |  |
| thiS8 | CGAAATTTTACACCCAAGGTGGTGTACCCCTTCGTGTGACCTTCACACTCTGTCCTATAGTGAGTCGTATTA | The template for transcription of M3 53 *thiS* RNA RNA |  |
| thiS9 | ACGAAATTTTACACCCAAGGTGGTGTACCCCTTCGTGTGACCTTCACACTCTGTCCTATAGTGAGTCGTATTA | The template for transcription of M3 54 *thiS* RNA RNA |  |
| thiS10 | GAAATTTTACACCCAAGGTGGTGTACGCCTTCGTGTGACCTTCACACTCTGTCCTATAGTGAGTCGTATTA | The template for transcription of M7 RNA |  |
| thiS11 | GAAATTTTACACCCAAGGTGGTGTATCCCTTCGTGTGACCTTCACACTCTGTCCTATAGTGAGTCGTATTA | The template for transcription of M8 RNA |  |
| thiS12 | GAAATTTTACACCCAAGCTGGTGTACCCCTTCGTGTGACCTTCACACTCTGTCCTATAGTGAGTCGTATTA | The template for transcription of M9 RNA |  |
| thiS13 | TAATACGACTCACTATA | The sense strand of T7 promoter used for *in vitro* transcription |  |
| thiS14 | TACGACGAATTCCAAAAATAATGTTGATCCTTTTAAATAAGTCTGATAAAATGTGAACTAATTTGACAGAGTGTGAAGGTCACACGAAGGGGTACACCACCTTGGGTGTATTTTTTCGTGTGGCCTTTTTTGCATGTATAAATAGGAAGAGGTGAAAAGTGGATCCAAAGGA | IDT G-block containing the wild-type *C.* sp. Maddingley *thiS* riboswitch and controlled by the *B. subtilis lysC* promoter, for cloning into pDG1661 as well as serving as a template for the dsDNA template for *in vitro* transcription termination |  |
| thiS15 | TACGACGAATTCCAAAAATAATGTTGATCCTTTTAAATAAGTC | Forward primer for the PCR amplification of the *C.* sp. Maddingley *thiS*  reporter construct |  |
| thiS16 | TCCTTTGGATCCACTTTTCACCTCTTCC | Reverse primer for the PCR amplification of the *C.* sp. Maddingley *thiS*  reporter construct |  |
| thiS17 | GTGAAGGTCACACGAAGGCGTACACCACCTTGGGTG | Forward primer for construction of the *C.* sp. Maddingley *thiS*  reporter as well as single-round transcription termination template containing M1 via QuikChange mutagenesis |  |
| thiS18 | CACCCAAGGTGGTGTACGCCTTCGTGTGACCTTCAC | Reverse primer for construction of the *C.* sp. Maddingley *thiS*  reporter as well as single-round transcription termination template containing M1 via QuikChange mutagenesis |  |
| thiS19 | CACACGAAGGGGTACACCAGCTTGGGTGTATTTTTTCG | Forward primer for construction of the *C.* sp. Maddingley *thiS*  reporter as well as single-round transcription termination template containing M2 via QuikChange mutagenesis |  |
| thiS20 | CGAAAAAATACACCCAAGCTGGTGTACCCCTTCGTGTG | Reverse primer for construction of the *C.* sp. Maddingley *thiS*  reporter as well as single-round transcription termination template containing M2 via QuikChange mutagenesis |  |
| thiS21 | GTACACCACCTTGGGTGTAAAATTTCGTGTGGCCTTTTTTG | Forward primer for construction of the *C.* sp. Maddingley *thiS*  reporter as well as single-round transcription termination template containing M3 via QuikChange mutagenesis |  |
| thiS22 | CAAAAAAGGCCACACGAAATTTTACACCCAAGGTGGTGTAC | Reverse primer for construction of the *C.* sp. Maddingley *thiS*  reporter as well as single-round transcription termination template containing M3 via QuikChange mutagenesis |  |
| thiS23 | CTTGGGTGTATTTTTTCCACTGGCCTTTTTTGCATG | Forward primer for construction of the *C.* sp. Maddingley *thiS* single-round transcription termination template containing M4 via QuikChange mutagenesis |  |
| thiS24 | CATGCAAAAAAGGCCAGTGGAAAAAATACACCCAAG | Reverse primer for construction of the *C.* sp. Maddingley *thiS*  reporter as well as single-round transcription termination template containing M4 via QuikChange mutagenesis |  |
| thiS25 | CAAAAATAATGTTGATCCTTTTAAATAAGTCTGATAAAATGTGAACTAATAGATATTTGACAGAGTGTGAAGGTC | Forward primer for the PCR amplification of the *C.* sp. Maddingley *thiS*  IVT template |  |
| thiS26 | ATCCACTTTTCACCTCTTCCTATTTATAC | Reverse primer for the PCR amplification of the *C.* sp. Maddingley *thiS*  IVT template |  |
| thiS27 | AAAAAAGGCCACACGAAAAAATACACCC | Reverse primer for the PCR amplification of the *C.* sp. Maddingley *thiS*  M5 IVT template |  |
| thiS28 | TACGACGAATTCCAACAGTAATGTTGATCCTTTTAAATAAGTCTGATAAAATGTGAACTAAATAAAATTTGATATAAATTGCAGGGGAACTAATTATAGTTGAGAAGGATAAAACCTGACCCTTAGAACCTGTCAGTTAATACTGTCGTAGGGAGCAAAGCACTGTGATTAAGATAAGATAAAGATGCTTTAATCTGTACGATTGAAGCATCTTTTTTAAATTTAATATTAATTAATCAGATTTTCAGTTTAAAGTAGGAAAATTTGAAACAGAAAACTTAATAAGTAACACGTAACTTTTATGTGAATTTAGAAAAGCATAAAAAGGGGTGCACCACCGCGGGTGTATCTACTTTTTATGCTTTTTTATTTAAAATTTAATAGATCAGACTTGGATCCAGCTGCA | IDT G-block containing the *C. lundense* tandem reporter controlled by the *B. subtilis lysC* promoter for cloning into pDG1661. |  |
| thiS29 | TACGACGAATTCCAACAGTAATGTTGATCCTTTTAAATAAGTCTGATAAAATGTGAACTAAATAAAATTTGATATAAATTGCAGGGGAACTAATTATAGTTGAGAAGGATAAAACCTGACCCTTAGAACCTGTCAGTTAATACTGTCGTAGGGAGCAAAGCACTGTGATTAAGATAAGATAAAGATGCTTTAATCTGTACGATTGAAGCATCTTTTTTAAATTTAATATTAATTAATCAGATTTTCAGTTTAAAGTAGGAAAATTTGAAACAGAAAACTTAATAAGTAACACGTAACTTTTATGTGAATTTAGAAAAGCATAAAAAGGCGTGCACCACCGCGGGTGTATCTACTTTTTATGCTTTTTTATTTAAAATTTAATAGATCAGACTTGGATCCAGCTGCA | IDT G-block containing the M1 *C. lundense* tandem reporter controlled by the *B. subtilis lysC* promoter for cloning into pDG1661. |  |
| thiS30 | TACGACGAATTCCAACAGTAATGTTGATCCTTTTAAATAAGTCTGATAAAATGTGAACTAAATAAAATTTGATATAAATTGCAGGGGAACTAATTATAGTTCTGAAGGATAAAACCTGACCCTTAGAACCTGTCAGTTAATACTGTCGTAGGGAGCAAAGCACTGTGATTAAGATAAGATAAAGATGCTTTAATCTGTACGATTGAAGCATCTTTTTTAAATTTAATATTAATTAATCAGATTTTCAGTTTAAAGTAGGAAAATTTGAAACAGAAAACTTAATAAGTAACACGTAACTTTTATGTGAATTTAGAAAAGCATAAAAAGGGGTGCACCACCGCGGGTGTATCTACTTTTTATGCTTTTTTATTTAAAATTTAATAGATCAGACTTGGATCCAGCTGCA | IDT G-block containing the M10 *C. lundense* tandem reporter controlled by the *B. subtilis lysC* promoter for cloning into pDG1661. |  |
| thiS31 | TACGACGAATTCCAACAGTAATGTTGATCCTTTTAAATAAGTCTGATAAAATGTGAACTAAATAAAATTTGATATAAATTGCAGGGGAACTAATTATAGTTCTGAAGGATAAAACCTGACCCTTAGAACCTGTCAGTTAATACTGTCGTAGGGAGCAAAGCACTGTGATTAAGATAAGATAAAGATGCTTTAATCTGTACGATTGAAGCATCTTTTTTAAATTTAATATTAATTAATCAGATTTTCAGTTTAAAGTAGGAAAATTTGAAACAGAAAACTTAATAAGTAACACGTAACTTTTATGTGAATTTAGAAAAGCATAAAAAGGCGTGCACCACCGCGGGTGTATCTACTTTTTATGCTTTTTTATTTAAAATTTAATAGATCAGACTTGGATCCAGCTGCA | IDT G-block containing the M11 *C. lundense* tandem reporter controlled by the *B. subtilis lysC* promoter for cloning into pDG1661. |  |
| thiS32 | TACGACGAATTCCAACAGTAATGTTGATCC | Forward primer for the PCR amplification of the *C. lundense* tandem reporter construct |  |
| thiS33 | TGCAGCTGGATCCAAGTCTGATCTATT | Reverse primer for the PCR amplification of the *C. lundense* tandem reporter construct |  |
